# Ethyl Acetate Extract from *Celastrus aculeatus* Merr. Suppresses Synovial Inflammation in Adjuvant Arthritis Rats through Apoptosis Induction of CD4^+^CD25^+^FOXP3^+^ T Cells

**DOI:** 10.1155/2014/460136

**Published:** 2014-12-08

**Authors:** Shu-tong Bai, Pei-hong Chen, Yu-yao Chen, Xiao-chun Lin, Jun-shan Liu, Li Tong

**Affiliations:** School of Traditional Chinese Medicine, Southern Medical University, Guangzhou 510515, China

## Abstract

*Celastrus aculeatus* Merr. has been widely used in traditional Chinese medicine to treat rheumatoid arthritis (RA) in clinic. However, the main active fraction of this plant is still unclear. In this study, we attempted to evaluate the suppressive effect of ethyl acetate extract (EAE) from *Celastrus aculeatus* Merr. on synovial inflammation in adjuvant arthritis (AA) rats induced by *Mycobacterium tuberculosis* H37Ra (Mtb) and to explore the underlying mechanisms. SD rats immunized with heat-killed Mtb were fed with EAE and observed for erythema, swelling, and induration of each paw. The pathologic changes in joint synovium were tested by hematoxylin-eosin staining. Apoptosis induction of synoviocytes was tested immunohistochemically. Apoptosis of peripheral lymphocytes and the level of regulatory T cells were analyzed by flow cytometry. After treatment with EAE, the joint inflammation in rats with AA was alleviated. Both apoptotic ratios of synoviocytes and peripheral lymphocytes and the ratio of CD4^+^CD25^+^FOXP3^+^ to CD4 regulatory T cells were significantly increased. In summary, we first demonstrated that EAE of *Celastrus aculeatus* Merr. can inhibit synovial inflammation in AA rats through apoptosis induction of CD4^+^CD25^+^FOXP3^+^ T cells. Our study provides a rationale for the application of *Celastrus aculeatus* Merr. to treat RA.

## 1. Introduction

Rheumatoid arthritis (RA) is a chronic autoimmune disease characterized by the inflammatory proliferation of synovial tissues and progressive destruction of articular cartilage. It was reported that the accumulation of inflammatory cells, proliferation of synovial tissue, and bone destruction in joints are related to immune network imbalance [1, 2]. Pathogenic effector T cells and regulatory T cells play a critical role in immune system, and the imbalance of pathogenic effector T cells and regulatory T cells is a predominant pathological feature of RA. A clinical trial indicated that the CD4 T cell subset percentage was increased in patients with RA, which competitively inhibited the proliferation of normal T cells and induced a significant decrease in the diversity of T cell clones [3]. Recently, regulatory T cells (Treg), which account for about 5%–10% of CD4^+^ T cells, have attracted much attention for its probable role in the development and progression of RA. The CD4^+^CD25^+^ T cells show immune regulatory functions both* in vitro* and* in vivo*, as they can inhibit the autoimmune diseases and may participate in the induction of transplantation tolerance. Therefore, CD4^+^CD25^+^ T cells play an important role in maintaining the stability of the internal environment [4], while the decrease or dysfunction of Treg may result in autoimmune diseases. Furthermore, Treg may slow down the progression of RA by regulating apoptosis of synovial cells and T lymphocytes [4, 5]. In China,* Celastrus aculeatus* Merr. has been traditionally applied to treat wind-damp Bi syndromes as this plant is believed to expel wind, eliminate dampness and swelling, promote Qi flow, invigorate blood, and detoxify the body. Clinically,* Celastrus aculeatus* Merr. is applied alone or in combination with other Chinese medicines (such as* Spatholobus suberectus* Dunn.) to treat arthritis and rheumatoid arthritis [6–8]. In previous studies, we demonstrated that the ethanol extract of* Celastrus aculeatus* Merr. has significant anti-inflammatory and analgesic effects in animal models [9–12] and suppresses the induction and progression of adjuvant arthritis (AA) induced by* Mycobacterium tuberculosis* H37Ra (Mtb) by modulating the immune response to heat-shock protein [13]. Our preliminary screening results also demonstrated that the ethyl acetate extract (EAE) from* Celastrus aculeatus* Merr. had better anti-inflammatory effects* in vitro* than the ethanol extract, suggesting the potential of EAE in the treatment of RA. In this study, we attempted to determine the effect of EAE on synovial inflammation in Sprague-Dawley (SD) rats with Mtb-induced AA and try to explore the underlying mechanisms of this action.

## 2. Materials and Methods

### 2.1. Animals

Male SPF SD rats (6–8 weeks old) were provided by the Experimental Animal Center of Southern Medical University (Guangzhou, China) and maintained under clean and well-ventilated conditions, with a room temperature of (23 ± 10)°C, humidity of (50 ± 5)%, and light-dark cycle of 12 : 12 h. The weights of SD rats were between 260 and 300 g. Research procedures were approved by the Laboratory Animals Care and Use Committee of Southern Medical University.

### 2.2. Medicines and Reagents

The roots and stems of* Celastrus aculeatus* Merr. were provided by the South China Institute of Botany in the Chinese Academy of Sciences (Guangzhou, China) and authenticated by Professor Hua-Gu Ye. Heat-killed Mtb was purchased from Difco (MD, USA), while methotrexate (MTX) injection was from Ebewe Pharma (Unterach, Austria) and erythrocyte lysate was from Andybio (IL, USA). Annexin V-FITC apoptosis kit, TUNEL apoptosis kit, and hematoxylin-eosin (HE) staining kit were procured from KGI (Nanjing, China). Lymphocyte separation medium was obtained from Haoyang (Tianjin, China). All other chemicals were purchased from Sigma (St. Louis, MO).

### 2.3. Preparation of EAE

Roots and stems of* Celastrus aculeatus* Merr. were dried and powdered. The powder was extracted thrice with 95% ethanol (RT, 1 h) and then concentrated to yield a residue. The residue was subsequently mixed with diatomite, placed in Soxhlet extractor, and extracted with ethyl acetate (ethyl acetate: residue = 20 : 1) for 7 h to obtain EAE. The yield of EAE was 59.7%.

### 2.4. Establishment of Animal Models and Their Grouping

SD rats were randomly divided into 5 groups with 12 rats in each group. The first group served as the control group and given normal saline by gavage once a day. The rest of the rats were injected with Mtb (1.0 mg/rat) in mineral oil subcutaneously at the base of the tail to establish AA animal models. The second group (model group) was given normal saline by gavage once a day. The third group (EAE low dose group) and the forth group (EAE high dose group) were treated with EAE (200 mg/kg or 400 mg/kg) by gavage daily after Mtb injection, respectively. The fifth group (MTX group) was fed with MTX (dosage of 6 mg/kg wt.) once per week. The total administration lasted for 4 weeks. Food intake and behavior of the rats were carefully observed, and the body weights were measured every seven days. After 7 days, the paw volume of right hind paw of rat was tested by 520 Plethysmometer Meter (IITC Life Science) and observed regularly for clinical signs of arthritis such as erythema, swelling, and induration. The severity of arthritis in each paw was graded on a scale from 0 to 4. The maximum arthritis score for each paw was 4, and the total arthritis score per rat was 16 [13].

### 2.5. Measurement of Blood Routine and the Rheological Properties of Blood

At the end of the experiments, SD rats were anesthetized by 10% chloral hydrate and abdominal aortic blood was taken. Then, 0.01% heparin sodium (v/v) was added to the blood and blood routine and the rheological properties of blood were detected by haematology analyzer (Hemavet 950FS, Drew, USA) and blood rheometer (LG-R-80, Shidi, China), respectively.

### 2.6. Pathologic Observation and Immunohistochemistry Analysis of the Knee Synovium in Rats with AA

At day 16 and day 28, 10% chloral hydrate was administrated to anesthetize rats from each group, respectively. Then, these rats were sacrificed and the knee synoviums of their hind limbs were harvested and fixated in formalin for 24 h. After paraffin embedding, sectioning at 5 *μ*m thickness was made, and each section underwent HE and immunohistochemistry analysis. The synovium stained by HE was observed under a microscope (Olympus) to detect synoviocyte proliferation and degeneration, inflammatory cell infiltration, and granulation tissue formation. The immunohistochemistry analysis was performed according to the manufacture's protocol to examine apoptotic synoviocytes. Cells with yellowish brown or dark brown cytoplasm were counted as apoptotic cells. The area with a positive expression and integrated optical density (IOD) was measured and analysed using the image analysis software package Image-Pro Plus 6.0. Synoviocyte apoptosis was reflected by the mean IOD (MIOD), calculated by the following formula: MIOD = IOD/area.

### 2.7. Apoptosis Analysis of T Lymphocytes in the Peripheral Blood of Rats with AA

At days 16 and 28, 10% chloral hydrate was administrated to anesthetize the SD rats. Blood samples collected from the abdominal aorta were mixed with Hanks' solution at a ratio of 1 : 1. The mixture was added to the lymphocyte separation medium. After centrifugation, the isolated T lymphocytes were stained by Annexin V-FITC/PI dyes, and flow cytometry (FACSCalibur, BD) was used to analyze the apoptotic rate of lymphocytes in the peripheral blood.

### 2.8. Determination of Regulatory T-Lymphocyte Level in the Peripheral Blood of Rats with AA

PECYy7-CD4 and FITC-CD25 antibodies were added into the blood samples from the abdominal aorta in the presence of heparin sodium for T cell subsets staining. Then, the mixture was protected from light at room temperature for 20 min, followed by the addition of 2 mL erythrocyte lysate. After 10 min, the mixture was centrifuged and the supernatant was discarded. About 1 mL of human fork head transcription factor 3 (FOXP3) fix buffer was added into the pellet and incubated for 20 min at room temperature. The mixture was centrifuged and washed twice with FOXP3 Fix Perm buffer. PE-FOXP3 antibody was added into the samples and incubated for 30 min at room temperature. The level of Treg in the peripheral blood was assessed by flow cytometry (FACSCalibur, BD).

### 2.9. Statistical Analysis

The data were expressed as the means ± SEM and analyzed by SPSS 13.0 software package (IL, USA). For statistical comparison, one-way ANOVA followed by Tukey's* post hoc* test was used and *P* < 0.05 was considered statistically significant.

## 3. Results

### 3.1. Inhibitory Effects of EAE on Mtb-Induced AA in Rats

**Figure 1:**
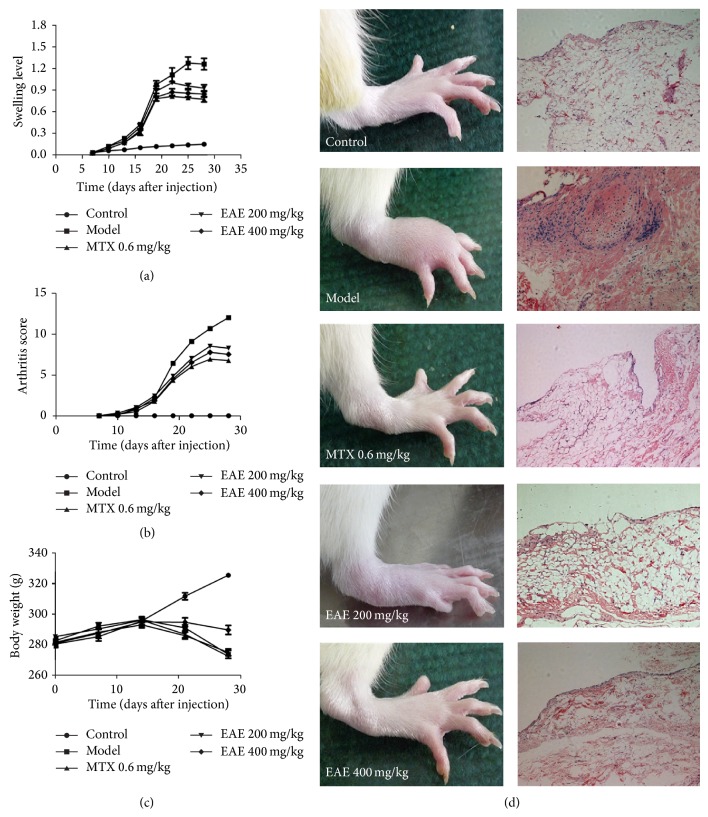
*In vivo* anti-inflammation effects of EAE in AA rats induced by Mtb. (a) Effects of EAE on the swelling level of paw in AA rats. 7 days after Mtb injection, the right hind paw volume of rat was detected every three days and arthritis swelling degree was calculated. (b) The arthritis score on paw swelling in AA model induced by Mtb in the presence or absence of EAE treatment. 7 days after Mtb injection, severity of arthritis in each paw was detected every three days and arthritic score was calculated. (c) Effects of EAE on body weight in AA rats induced by Mtb. After 14 days of injection, the body weight of each group was measured every seven days. (d) Effects of EAE on clinical and histological changes of ankle joints. At day 28, rats were sacrificed. The knee synoviums of their hind limbs were harvested and fixated. After paraffin embedding and sectioning at 5 *μ*m thickness, the knee synoviums were stained by hematoxylin and eosin (H&E) and observed under a microscope.

During experiment period, autonomic activities of rats in the control group were normal, without any abnormalities in food intake or behavior. The joints of rats in the control group were soft without any erythema and swelling. However, from day 10, erythema and swelling in toes and ankles of rats after Mtb injection were observed (Figure 1(a)), accompanied by depression and food intake reduction. Part of rats had tail nodules or ear erythema. The level of swelling peaked at day 25, and arthrosclerosis and limp appeared in some rats. The body weights of rats were significantly decreased after 14 days. However, these phenomena were relieved after EAE treatment, especially at high dose treatment (Figures 1(b) and 1(c)). Pathological results (Figure 1(d)) showed that the synovial tissues of rats in the control group were smooth and thin and synoviocytes were flattened without any inflammatory cell infiltrations. However, the abnormal hyperplasia was identified in synovial tissues of rats in the model group. There were a large number of inflammatory cells infiltrations in the synovium, accompanied with the formation of granulation tissue. After drug treatment, both hyperplasia of synovial tissues and infiltrations of inflammatory cells were attenuated in a dose-dependent manner, suggesting the protective effect of EAE on the joint synovium. To detect the influence of EAE on blood physiology of rats, blood parameters were analysed. As shown in Table 1, white blood cells (WBC) and platelet (PLT) count and hematocrit (HCT) were elevated, while the concentration of haemoglobin B (Hb) was decreased in the model group compared with the control group. Besides, lymphocyte (LY), monocyte (MO), and red blood cell (RBC) count did not change obviously, while the abnormality in blood hemogram was relived after EAE injection. We further analyzed the change of the rheological properties of blood by blood rheometer (LG-R-80, Shidi, Beijing). Mtb injection could increase the degree of blood viscosity (BV) and plasma viscosity (PV). However, after EAE injection, the blood parameters were not only decreased, but were also less than those in the control group (Table 2). These results implied that EAE can relieve the abnormality of blood physiology in AA rats.

### 3.2. EAE Treatment Induces Synoviocyte Apoptosis in the Joints of Rats with AA

At day 16 and day 28, after Mtb injection, the apoptosis of synoviocytes in the joints of SD rats with AA was observed under a microscope (Olympus), and the apoptotic cells were counted. As shown in Table 3, the number of apoptotic synoviocytes in the model group was decreased compared to the control group, demonstrating the deficiency of synoviocytes apoptosis in the joints of SD rats with AA. After 16-day or 28-day treatment of MTX or EAE, the apoptotic rate of synoviocytes was increased significantly. Moreover, the proapoptotic effects of EAE in the high dose group on synoviocytes in AA rats were almost similar to those of MTX.

### 3.3. EAE Treatment Induces Peripheral T Lymphocyte Apoptosis in Rats with AA

**Figure 2:**
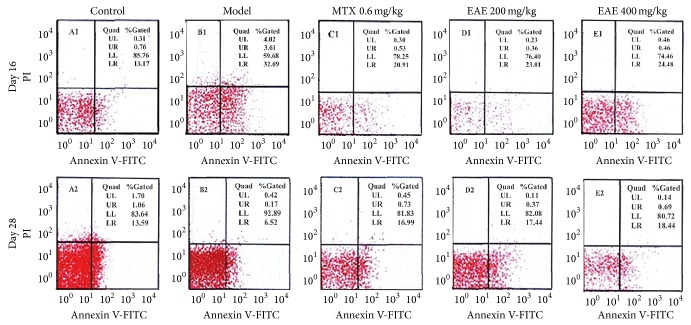
Effects of EAE on apoptosis of peripheral T lymphocytes in AA rats induced by Mtb. At days 16 and 28, T lymphocytes were isolated from abdominal aorta and stained by Annexin V-FITC/PI dyes. The apoptotic rate of lymphocytes was analyzed by flow cytometry.

At day 16, after Mtb immunization, the apoptotic rate of peripheral lymphocytes in the model group was increased obviously compared with that of peripheral lymphocytes in the control group. However, at day 28, after Mtb injection, the number of apoptotic peripheral lymphocytes was decreased significantly (Table 3 and Figure 2). These results suggested that lymphocytic apoptosis is changed during the process of joint inflammation. In the EAE-treated groups, the apoptotic rate of peripheral lymphocytes dropped, at day 16, but increased at day 28 (Table 4), compared with the model group. These results indicated that EAE treatment may protect peripheral lymphocytes from abnormal apoptosis to maintain the life or death balance of peripheral lymphocytes.

### 3.4. Effects of EAE on CD4 T Cell Count, CD25 T Cell Count, and FOXP3 Level in Rats with AA

**Figure 3:**
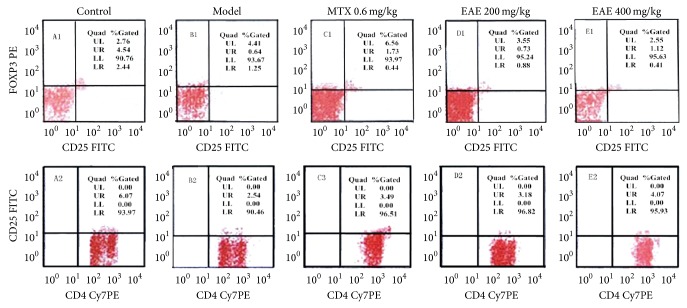
Effects of EAE on peripheral regulatory T cells in AA rats induced by Mtb. After 28 days, rats were sacrificed. Blood from the abdominal aorta was incubated with PECy7-CD4, FITC-CD25, and PE-FOXP3 antibodies. The proportion of CD4^+^CD25^+^FOXP3^+^ T cells and CD4^+^CD25^+^ T cells in peripheral blood CD4^+^ T cells was detected by flow cytometry.

The flow cytometry data (Table 5 and Figure 3) showed that, at day 28 after Mtb injection, the percentage of both CD4^+^CD25^+^FOXP3^+^ regulatory T cells in CD4 T cells and the percentage of CD4^+^CD25^+^ T cells in the total CD4 T cells in the model group were reduced compared to the control group. The data indicated that peripheral regulatory T cells at the advanced stage of AA are deficient. However, EAE treatment significantly increased the level of peripheral regulatory T cells in rats with Mtb-induced AA. The percentages of CD4^+^CD25^+^FOXP3^+^ and CD4^+^CD25^+^ regulatory T cells in CD4^+^ T cells were increased from 0.54 ± 0.20% to 1.76 ± 0.17% and from 2.59 ± 0.34% to 4.51 ± 1.17% at 400 mg/kg, respectively.

## 4. Discussion

RA is a frequently occurring human disease, which peaks at 20–45 years, and the male-to-female incidence ratio is about 1 : 3. RA may lead to persistent synovitis, progressive joint damage and dysfunction, and even disabilities. The occurrence of these phenomena causes great pain and heavy burden to the patients and families [1, 2]. RA is mainly caused by hereditary, infection, and hormones abnormalities [14, 15]. With the advancement of scientific studies on the pathogenesis of RA, the treatments for RA are focused on drug therapy, rehabilitation, surgical treatment management, diet, and psychotherapy. Among them, drug therapy is most frequently used in clinic. Anti-RA drugs mainly include nonsteroidal anti-inflammatory drugs (NSAID), diseases-modifying antirheumatic drugs (DMARD), hormones, and some natural agents [15]. However, traditional RA drugs have high toxicity, while biological and gene therapy is expensive and poor compliance to patients. Therefore, it is urgent to study the anti-RA drugs with high efficiency and low toxicity.* Celastrus aculeatus* Merr. is used to treat arthritis and rheumatoid arthritis in China [6–8]. Our previous studies have demonstrated that the ethanol extract of* Celastrus aculeatus* Merr. has significant anti-inflammatory effects in AA rat models [10, 13]. We also found that EAE had better anti-inflammatory effects* in vitro* than the ethanol extract. Thus, we evaluated the* in vivo* activity of EAE in AA rat models. Our results showed that the degree of paw swelling and inflammatory score of rats after EAE injection was significantly reduced in EAE groups compared with the model group, which suggested that EAE has good inhibitory effects on joint inflammation of AA rats. The microscopic observation of synovium of joint further improved as EAE could obviously suppress the synovial hyperplasia, the infiltration of inflammatory cells, and the formation of granulation tissue in AA rats. We also noticed that EAE treatment at 400 mg/kg could significantly relieve the decrease of body weight in AA rats compared with MTX group. Besides, no abnormal activities and food intakes were observed in EAE groups, indicating that the toxicity of EAE was lower than that of MTX.

Clinical studies reported that most of the patients with RA have abnormal blood parameters with anaemia and increased WBC count that may be reduced due to the appearance of severe anaemia [16–18]. Thrombocytosis was found to be 1 to 2 times higher than normal [18]. On the other hand, the viscosity of blood and plasma, the concentrations of fibrinogen and immunoglobulin, and the erythrocyte sedimentation rate (ESR) are increased, with no change in hematocrit value [19]. In AA model, Mtb induces destructive arthritis through the interaction with immune cells by the abundant release of inflammatory factors (tumor necrosis factor *α*, INF-*γ*, interleukin 1, and interleukin 6). These inflammatory factors influence the hematopoietic function of erythroid cells, the activation of platelets, and the increase of fibrinogen and immune globulin, leading to the hematological change [20, 21]. In this study, we found that the total numbers of WBC, LY, and MO in AA rats were similar to the normal group. However, the haemoglobin content was decreased, with increased platelet, hematocrit, BV, and PV, similar to that of RA patients. Interestingly, the EAE treatment reversed these abnormalities, implying that this extract can improve the abnormal changes of the rheological properties of blood in RA process. Thus, our study demonstrated that EAE may decrease the symptoms and improve the joint function by regulation of blood parameters. Since this haematology study only performed on 28-day samples which presented the late hematologic change, but not the changes in the whole course of EAE treatment, further study was required.

The pathogenesis of RA is associated with abnormal apoptotic process, including synovial cells, fibroblasts, lymphocytes, and cartilage cells. Some earlier study showed that the apoptosis deficiency of synovial cells lead to synovial cells proliferation, which is an important pathological mechanism on RA morbidity and progression [22], as synovial cells are composed of RA synovial fibroblasts (RA-SF), scavenger synovial cells, and dendritic cells like synovial cells. Among them, RA-SF is accounted for 70% of synovial cells [1, 22]. Currently, more and more convincing evidence demonstrated that RA-SF play a critical role in RA pathogenesis by participating in the destruction of cartilago articularis. Moreover, it was also found that, with the exacerbation of joint inflammation, the joint destruction was aggravating in the AA rats, accompanied by the attenuation of RA-SF apoptosis. These results suggested that there is a correlation between RA-SF apoptosis and the severe degree of joint arthritis [1, 23, 24]. Our study demonstrated that, after Mtb immunization, the level of synovial cell apoptosis in the joints of rats with AA was decreased, compared to the rats in the control group, indicating that there is the deficiency of synovial cells apoptosis in the joints of AA rats. However, after EAE treatment, the apoptotic level of synovial cells in the joints of AA rats was significantly increased, demonstrating the regulation effects of EAE on synoviocyte apoptosis in the joints of rats with AA.

Recent results showed that the peripheral blood lymphocytes in RA patients are significantly less due to apoptosis, depending on the degree of RA [25, 26]. We found that apoptosis rate of peripheral blood lymphocytes in AA rats was raised in the middle of the treatment course and decreased in the later period. It may be that peripheral blood lymphocytes were proliferative significantly, in the middle of the treatment course. At the same time, compensatory apoptosis of cells was increased. And, at the late stage, the apoptosis rate of lymphocytes was dropped to a lower level. However, EAE treatment could promote lymphocyte apoptosis. Therefore, we concluded that, throughout the course, abnormal lymphocyte apoptosis induces the release of inflammatory factors to mediate the interactions of lymphocytes, which caused the occurrence and development of RA, while EAE played a regulatory role in the process.

RA is a T cell mediated autoimmune disease, and CD4^+^ T cells play an important role in the process of RA. The percentage of CD4^+^ T cell subgroup in RA patients is increased to competitively inhibit the proliferation of normal T cell, leading to the reduction of the diversity of T clone cells [3]. Treg, a type of T cells that can prevent the autoimmune response and maintain immune balance, attracted more attention in recent years, because of their critical role in the occurrence and development of RA [4]. Until now, a variety of phenotypic Treg, including CD4^+^ CD25^+^ Treg, type 1 regulatory T cell (Tr 1), and helper T cells (Th3**),** have been discovered. CD4^+^ CD25^+^ Treg are a major component of Treg subtypes which are found in the peripheral blood and spleen of mice or human and accounted for about 5%–20% of CD4^+^ T cells [25]. The decrease or dysfunction of Treg may result in autoimmune disease [26]. Clinical evidence suggested that CD4^+^ CD25^+^ Treg were abnormal in RA patients and the level of Treg in the peripheral blood of RA patients was fluctuated at different periods [27, 28]. Moreover, transcription factor FOXP3, specifically expressed in CD4^+^ CD25^+^ Treg, is recognized as the specific biomarker. Several evidences have suggested that the proportion of CD4^+^ CD25^+^ FOXP3^+^ Treg in peripheral blood of RA patients was reduced significantly along with the mRNA and protein levels of FoxP3 in RA patients [29]. Furthermore, the expression of FOXP3 plays an important role in regulating the development and function of Treg and is closely related to autoimmune disease. Thus, Treg expressing FOXP3 are proposed to link to the pathological progress of RA [30]. We observed that at day 28, after Mtb immunization of the SD rats, both CD4^+^ CD25^+^ FOXP3^+^ Treg and CD4^+^ CD25^+^ T cell subsets are reduced in the CD4^+^ T cells and the administration of EAE could significantly increase the percentage of Treg in rats with AA. These results indicated that EAE can suppress inflammation in AA rats by regulating Treg to influence immunity.

In a summary, we for the first time demonstrated that EAE from* Celastrus aculeatus* Merr. can suppress the joint inflammation of rats with AA through apoptosis induction of synoviocytes in the joints and peripheral T lymphocytes, associated with the regulation of CD4^+^ CD25^+^ FOXP3^+^ Treg. Our results provide a rationale for the clinical use of* Celastrus aculeatus* Merr. However, the anti-inflammatory mechanisms of EAE need to be further explored.

## Figures and Tables

**Table 1:** Effects of EAE on blood routine in AA rats.

Group	WBC (K/*μ*L)	LY (K/*μ*L)	MO (K/*μ*L)	RBC (K/*μ*L)	Hb (M/*μ*L)	PLT (g/dL)	HCT (%)
Control	18.19 ± 4.53	4.64 ± 1.64	1.09 ± 0.35	8.52 ± 0.17	16.59 ± 0.33	962.17 ± 310.13	42.95 ± 3.01
Model	24.25 ± 5.3^*^	4.06 ± 0.96	1.56 ± 0.50	8.12 ± 0.46	15.55 ± 1.00^*^	1350.17 ± 131.16^*^	49.08 ± 0.95^*^
MTX 0.6 mg/kg	20.87 ± 4.7	5.78 ± 0.6	1.31 ± 0.32	8.27 ± 0.28	14.27 ± 0.96^*^	973.83 ± 146.19^#^	41.15 ± 3.67^#^
EAE 200 mg/kg	15.33 ± 2.29^#^	5.40 ± 1.12	1.15 ± 0.19	8.10 ± 0.49	16.30 ± 1.17	1047.17 ± 48.69^#^	44.20 ± 1.77^#^
EAE 400 mg/kg	14.70 ± 1.36^#^	5.31 ± 1.22	1.07 ± 0.18	8.47 ± 0.56	16.33 ± 0.59	1072.00 ± 249.87^#^	42.28 ± 2.90^#^

^*^ *P* < 0.05 versus control group; ^#^ *P* < 0.05 versus model group.

**Table 2:** Effects of EAE on blood rheology in AA rats.

Group	BV^150/s^ (mPas)	PV^100/s^ (mPas)
Control	6.94 ± 0.41	1.62 ± 0.04
Model	7.29 ± 0.56^*^	2.93 ± 065^*^
MTX 0.6 mg/kg	5.96 ± 0.81^∗#^	1.66 ± 0.05^#^
EAE 200 mg/kg	5.22 ± 0.84^∗#^	1.79 ± 0.09^∗#^
EAE 400 mg/kg	6.61 ± 0.58^#^	1.70 ± 0.06^#^

^*^ *P* < 0.05 versus control group; ^#^ *P* < 0.05 versus model group.

**Table 3:** Effects of EAE on synoviocyte apoptosis in AA rats.

Group	Synoviocyte apoptosis-MIOD (×10^−2^/*μ*m^2^)	
	Day 16	Day 28
Control	42.07 ± 2.35	41.93 ± 2.04
Model	24.09 ± 2.65^*^	19.75 ± 2.00^*^
MTX 0.6 mg/kg	50.88 ± 3.34^∗#^	44.44 ± 2.13^#^
EAE 200 mg/kg	44.08 ± 1.34^∗#^	38.32 ± 0.95^#^
EAE 400 mg/kg	51.79 ± 0.16^∗#^	44.03 ± 1.97^#^

^*^ *P* < 0.05 versus control group; ^#^ *P* < 0.05 versus model group.

**Table 4:** Effects of EAE on the peripheral T lymphocytic apoptosis rate in AA rats.

Group	Apoptotic rate (%)	
	Day 16	Day 28
Control	13.85 ± 0.92	13.42 ± 1.81
Model	34.49 ± 2.61^*^	6.46 ± 0.83^*^
MTX 0.6 mg/kg	20.46 ± 1.25^∗#^	16.07 ± 2.40^#^
EAE 200 mg/kg	21.33 ± 2.09^∗#^	16.37 ± 2.09^∗#^
EAE 400 mg/kg	23.13 ± 3.47^∗#^	17.27 ± 0.52^∗#^

^*^ *P* < 0.05 versus control group; ^#^ *P* < 0.05 versus model group.

**Table 5:** Effects of EAE on the level of peripheral regulatory T cells in AA rats.

Group	CD4^+^CD25^+^FOXP3^+^/CD4^+^ (%)	CD4^+^CD25^+^/CD4^+^ (%)
Control	3.71 ± 0.80	5.34 ± 0.64
Model	0.54 ± 0.20^*^	2.59 ± 0.34^*^
MTX 0.6 mg/kg	1.79 ± 0.09^∗#^	3.53 ± 0.39
EAE 200 mg/kg	0.75 ± 0.08^*^	3.05 ± 0.11
EAE 400 mg/kg	1.76 ± 0.17^∗#^	4.51 ± 1.17

^*^ *P* < 0.05 versus control group; ^#^ *P* < 0.05 versus model group.

## Abbreviations

EAE: Ethyl acetate extract
AA: Adjuvant arthritis
Mtb: * Mycobacterium tuberculosis* H37Ra
HE: Hematoxylin and eosin
RA: Rheumatoid arthritis
Treg: Regulatory T cells
SD: Sprague-Dawley
MTX: Methotrexate
IOD: Integrated optical density
MIOD: Mean IOD
FOXP3: Forkhead transcription factor 3
WBC: White blood cells
PLT: Platelets
HCT: Hematocrit
Hb: Haemoglobin B
LY: Lymphocyte
MO: Monocyte
RBC: Red blood cell
BV: Blood viscosity
PV: Plasma viscosity
NSAID: Nonsteroidal anti-inflammatory drugs
DMARD: Diseases-modifying antirheumatic drugs
ESR: Erythrocyte sedimentation rate
RA-SF: RA synovial fibroblasts.
